# Supplementary material for: Eprinomectin and Moxidectin Resistance of Trichostrongyloids on a Goat Farm in Austria
Source: Pathogens. 2022 Apr 21;11(5):498. doi: 10.3390/pathogens11050498 (PMC9143937; doi:10.3390/pathogens11050498)
Supplement: Supplementary file 1 [file pathogens-11-00498-s001.zip › pathogens-1696473-supplementary.pdf]

Table S1. Data on animals (m= male; f= female; Shz= Steirische Scheckenziege (Styrian piebald goat; a local mountain goat breed); Va= Valais black necked goat; EPN= eprinomectin; MOX= moxidectin; EpG= eggs per gram of faeces; bt= before treatment; pt= post treatment; - = no data).

Goat nr.	Age (years)*	Sex	Breed	EPN		MOX	
				EpG bt	Epg pt	EpG bt	Epg pt
1	5	m	Shz	1660	1910	-	-
2	6	f	Shz	0	-	450	35
3	4	f	Shz	0	-	2930	785
4	4	f	Shz	360	180	1410	140
5	4	f	Shz	0	-	1465	285
6	2	f	Shz	5	-	1520	125
7	2	f	Shz	1255	585	885	60
8	2	f	Shz	905	240	2165	15
10	0.5	f	Shz	565	455	1360	1155
11	1	f	Shz	475	1020	-	-
12	0.5	m	Shz	25	-	-	-
13	3	f	Shz	105	135	1555	70
14	3	f	Shz	0	-	1310	90
15	2	f	Shz	0	-	2375	5
16	3	f	Shz	45	-	-	-
17	2	f	Shz	10	-	-	-
18	2	f	Shz	1045	640	880	375
20	0.5	f	Shz	485	20	-	-
21	0.5	f	Shz	1230	445	-	-
22	0.5	f	Shz	765	30	-	-
19	0.5	f	Va	1540	315	2625	650
23	0.5	f	Va	740	325	2240	1270
24	10	f	Va	1140	1170	4430	105
25	6	m	Va	2125	4280	3950	1465
26	0.5	f	Va	1910	920	-	-
27	8	f	Va	0	-	4025	65
28	8	f	Va	1690	415	-	-
29	8	f	Va	0	-	-	-
